# The implications of the hypocitricemic response to surgery and the role of liver function and hepatocyte metabolism: An important, but neglected, clinical relationship

**DOI:** 10.15406/jlrdt.2018.04.00112

**Published:** 2018-06-08

**Authors:** Leslie C Costello, Renty B Franklin

**Affiliations:** Department of Oncology and Diagnostic Sciences, University of Maryland School of Dentistry, USA

## Abstract

Reported studies more than forty years ago established that all surgery patients exhibit a marked postoperative hypocitricemia within one day following surgery and persists for seven days and longer. Animals also exhibit the postoperative hypocitricemia. The hypocitricemia results from increased liver clearance of plasma citrate, in which the hepatocytes become capable of transporting and utilizing citrate from plasma. This represents a physiologic/metabolic response during the patient recovery from surgery. The extensive hypocitricemia in response to surgery is not manifested by known citricemic hormones, but is initiated via an unidentified putative endocrine hypocitricemic hormone. In addition to the importance relating to surgery patients, the surgical hypocitricemic effects, along with the liver and hepatic cell effects, will impact virtually all human and animal clinical and experimental studies that include surgical intervention; including the conclusions and translational clinical implications.

Unfortunately, the hypocitricemic response to surgery has been ignored for the past forty years, and most contemporary clinicians and biomedical investigators are not aware of this clinical relationship. The intent of this review is to inform members of the medical community of the established hypocitricemic response to surgery and the important role of liver clearance and hepatocyte metabolism of plasma citrate; which, hopefully, will generate interest and research that should be integrated into contemporary issues that involve surgical intervention.

## Introduction

The first report of hypocitricemia following surgery in humans is credited to Sjostrom in 1937.^1^ This was followed by a report of no change in plasma citrate,^2^ and a report of hypocitricemia following surgery.^3^ The issue was resolved by the studies of Costello et al studies^4–7^ over the period of 1971–1977, which conclusively established that all surgical patients and animals (dogs and rats) exhibit marked hypocitricemia following surgery. The studies also identified that increased liver clearance of citrate is the major mechanism resulting in the hypocitricemic response to surgery, which was a new role identified for liver function. Since those early studies, no other reports of surgical hypocitricemia exist (as best that we could establish); and based on a Google search, only about four reports have cited or made reference to the hypocitricemic effects of surgery. It is apparent that the existence of surgical hypocitricemia and its implications have virtually received no attention or recognition by the clinical and biomedical research community over the past forty years.

Plasma citrate is a homeostasis factor in humans and animals that is physiologically maintained within a normal range. A sustained plasma citrate concentration below or above this range presents hypocitricemia/hypercitricemia, which can be indicative of a pathophysiological and possible clinical condition. However, the persistent marked hypocitricemia following surgery does not result in associated pathophysiological consequences (such as hypocitraturia and nephrolithiasis; metabolic acidosis) during the postoperative recovery of patients. Thus, the hypocitricemia reflects the importance of increased liver clearance and hepatocyte uptake and metabolic utilization of plasma citrate, as an important physiological response to surgical stress.

It is highly important to recognize that the hypocitricemic physiological and metabolic responses to surgery will be manifested in essentially all clinical and experimental studies that involve surgical interventions, especially in regard to the induction of the liver and hepatocyte functional/metabolic relationships. This applies to the important contemporary clinical issues regarding the process of liver regeneration following conditions such as liver damage, liver transplant, and partial hepatectomy. In these and other such conditions, the hypocitricemic response to surgery is most likely involved along with the liver regeneration process; and must be recognized as a confounding factor that must be addressed. This has not been considered in any such contemporary clinical or experimental reports involving surgical interventions; thereby raising serious concerns about the appropriateness of the studies, and their results and conclusions.

This review intends to bring attention and describe the established hypocitricemic relationships following surgery in patients and animals. The impact of the factors and conditions of surgical hypocitricemia on the studies of the relationships of liver regeneration is described. This is essential to conduct appropriate clinical and experimental studies that will establish the correct relationships of issues that involve surgical intervention. It also calls attention to the need for more research to identify the endocrine and possible autocrine/paracrine factors and mechanisms that are involved in the liver citrate clearance response and the hepatocyte transport and metabolic utilization of plasma citrate.

## The homeostatic maintenance of plasma citrate concentration

An expanded background and description of citrate homeostasis is presented in our recent review,^8^ which contains additional relevant information. The following highlights some of the important relationships.

The homeostasis of normal plasma citrate concentration in humans is maintained within a range of ~100–150uM. Conditions that tend to increase or decrease the plasma citrate beyond this normal range will trigger physiological, endocrinological, and metabolic responses that restore and maintain the normal plasma citrate concentration. A sustained decreased or increased concentration beyond the normal range, presents a hypocitricemia or hypercitricemia that could be indicative of a pathophysiological status, which can impose clinical consequences. For example, hypercitricemia can decrease the plasma calcium ion concentration; thereby impairing blood clotting, neuromuscular activity, cardiac activity and other such effects. Hypocitricemia is associated with decreased citrate concentration of renal tubular fluid and urine; which is an important factor in kidney stone formation. Also, the acid-base status can be dependent on the plasma citrate concentration.

The maintenance of the normal plasma citrate concentration is achieved by the balance between citrate that is provided to circulation and its removal from circulation. Typically, dietary citrate and citrate from bone represent the major sources for plasma citrate. The removal of citrate from plasma is the role of the renal clearance of plasma citrate. Under special conditions, other factors can contribute to the source of plasma citrate (such as cellular production and secretion of citrate; or can contribute to the removal of citrate from plasma (such as transport of plasma citrate into cells). The maintenance is achieved by hypercitricemic hormones/factors (such as parathyroid hormone and vitamin D) and hypocitricemic hormones (such as calcitonin); i.e. hypercitricemic and hypocitricemic hormones and other systemic plasma factors.

## The hypocitricemic response in patients following surgery

Following our initial report^5^ that included 30 surgery patients, additional surgery patients were studied; which collectively totaled 65 surgery cases.^6^ Of this total, 63 cases exhibited the hypocitricemic response; and in the 2 remaining cases, the patients exhibited a pre-op hypocitricemia of undetermined origin that was sustained following surgery. Therefore, all surgical cases exhibited post-op hypocitricemia; and none of the cases exhibited normocitricemia or hypercitricemia following surgery.

**Figure 1:**
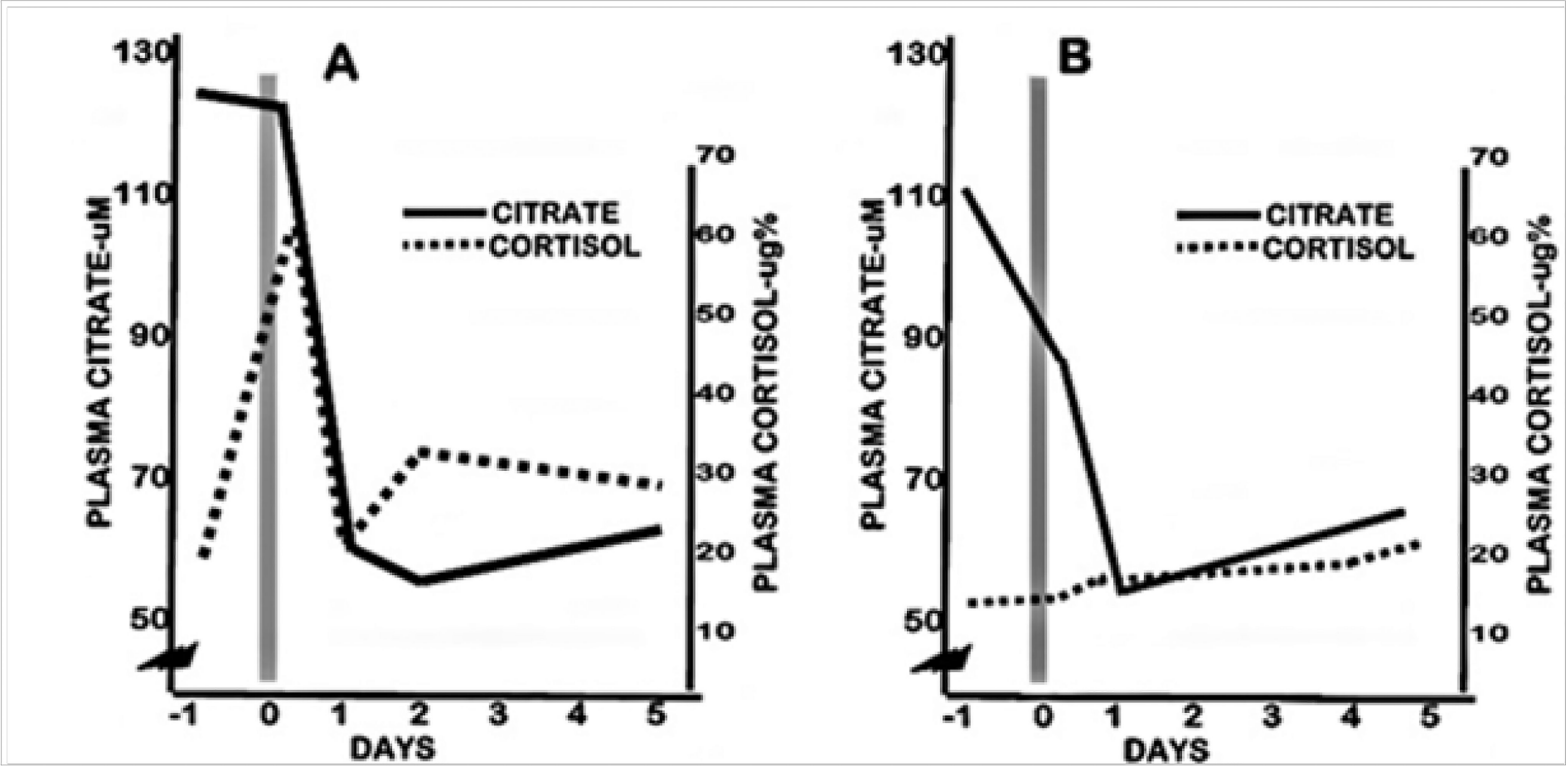
Representative cases of hypocitricemia following surgery in patients. A and B show typical hypocitricemia with different cortisol responses.

Table 1 is the composite of the 30 patients in this initial study,^5^ which reveals the significant decrease in plasma citrate within 1day following surgery and persists for at least 7days. The consistent hypocitricemia is seen in all surgical procedures, in male and female patients; in all ages (from 22–65year-old patients in this study); and other variables represented in the total patient population in these studies. Figure 1 (modified from^5^) presents two cases, which show the representative consistent hypocitricemia following surgery, even though the cortisol response is markedly different. Because most patients were released from hospital care by 7days post-op, these studies could not establish the extended period of hypocitricemia beyond this post-op period. However, a few available post-op subjects maintained the hypocitricemia for up to 2weeks.^5^

The cortisol response (Table 1) (Figure 1) revealed that cortisol did not correlate with the surgical hypocitricemia; and subsequent animal studies established that adrenal corticoids do not exhibit the surgical hypocitricemia effect.^5^ Since the actions of citricemic hormones (such as parathyroid hormone and calcitonin) are often coupled to their respective calcemic effects, Table 1 shows that hypocalcemia does not correlate with the hypocitricemia; and this is also revealed in the animal studies.^4,6^

**Figure 2:**
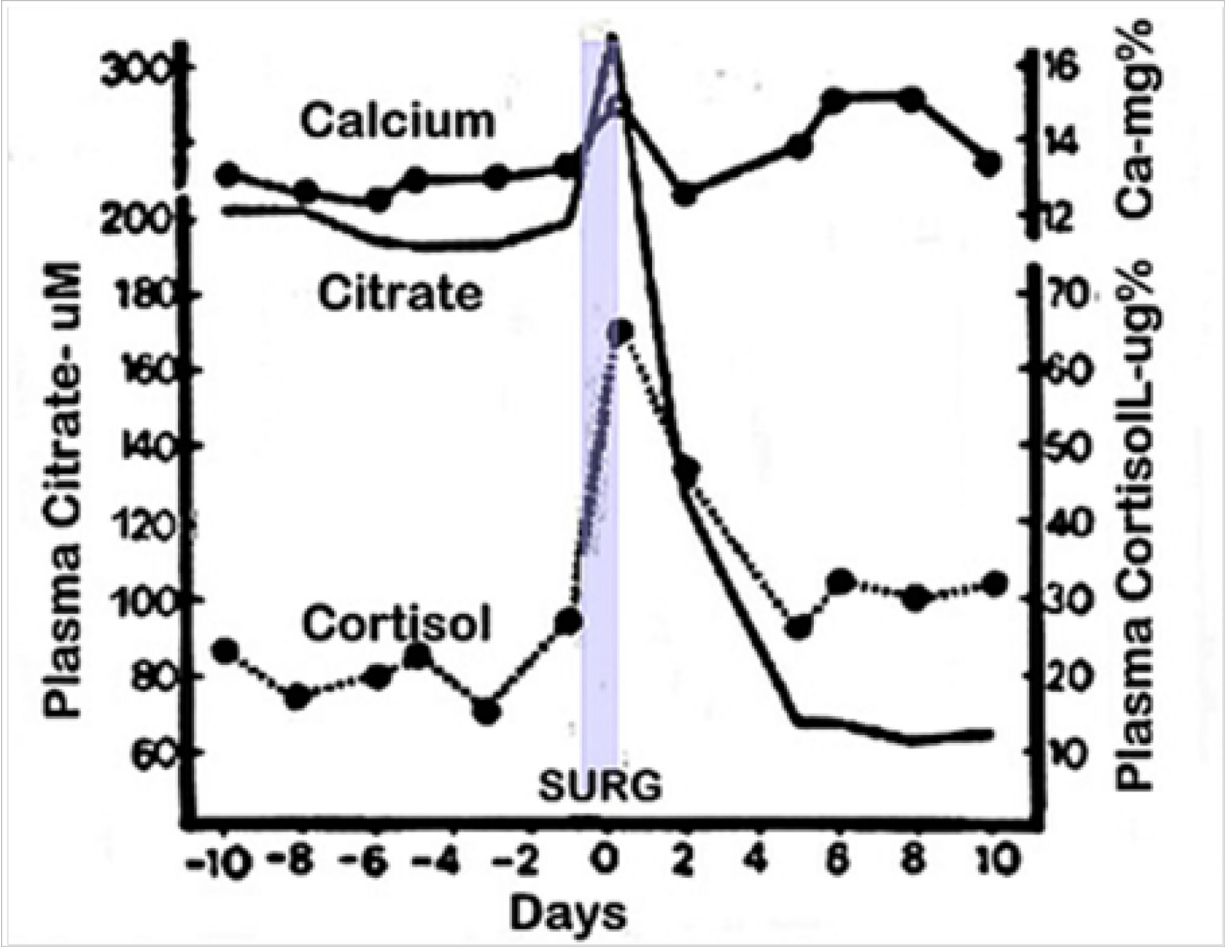
The hypocitricemic response following surgery in a hyperparathyroid patient. The patient presented with rectal carcinoma and primary hyperparathyroidism. A perineal resection was performed; and the patient was still hyperparathyroid following surgery.

Especially relevant is the hypocitricemic response in a patient who was diagnosed with hyperparathyroidism along with carcinoma of the rectum (Figure 2).^5^ The patient presented with severe hypercitricemia and hypercalcemia, which characterizes severe hyperparathyroidism. An abdominoperineal resection of the colon carcinoma was performed; which provided the opportunity to observe the surgical effects under conditions where the major hypercitricemic agent (PTH) prevailed. Immediately after surgery the hypercitricemia “spiked”; which was then followed by a rapid decrease in plasma citrate, culminating in severe persistent hypocitricemia for more than 10days. Thus, the hypocitricemic effect of surgery prevailed over the pronounced hypercitricemic effect of parathyroid hormone, so that the plasma citrate was decreased from ~300 to ~60uM. This is an extremely dominant hypocitricemic effect that results from surgery. The persistent hyperparathyroid status following surgery is evident by the sustained prevalent hypercalcemia during the post-op period. This is an extremely important revelation as it demonstrates a new understanding that the hypercitricemic effect of parathyroid hormone can be independent of its hypercalcemic action^8^ Also notable is the persistent post-op hypocitricemia although cortisol returned to normal concentration; which is consistent with our other studies. Thus, a dominant hypocitricemic agent is involved in the response to surgery.

**Figure 3:**
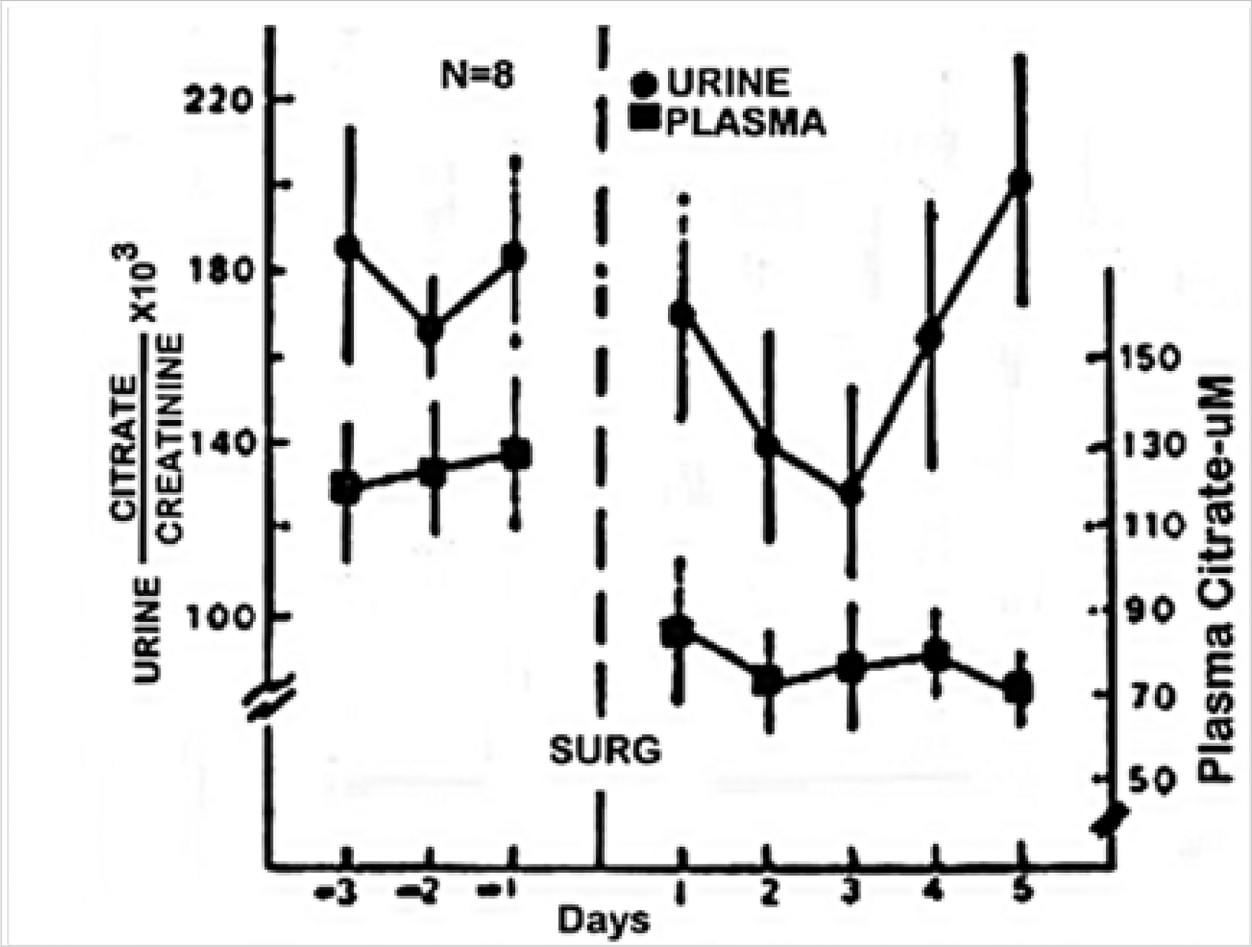
Surgery postoperative effects on patient plasma citrate concentration and urinary excretion of citrate. The citrate concentration was determined from 24-hours collections of urine prior to and following surgery of 8 patients.

The major physiological process that is typically involved in the manifestation of hypocitiricemia is increased renal clearance of citrate. However, Figure 3 demonstrates the absence of increased clearance and urinary excretion of citrate associated with the surgical hypocitricemia in patients.^5^ This was also evident in the animal studies,^6^ which revealed no change in the renal clearance of citrate (described below). Consequently, another process must be involved in manifesting the hypocitricemia following surgery; which we addressed in the animal studies.^6,7^

## The hypocitricemic response in animals following surgery

Studies with dogs^5^ demonstrated the hypocitricemia by 1day following surgery, which persists for ~4days; thereafter returning to normocitricemia. Control animals subjected to anesthesia only, maintained normocitricemia; thereby substantiating the hypocitricemia is a surgical event independent of anesthesia. Table 2 shows the hypocitricemia following laparotomy in rats.^6^ A difference between the animal (dog and rat) and the human hypocitricemic response is that the animals return to normocitricemia much more rapidly than humans. This is not due to differences in the surgical procedure since the procedures in the animal studies included splenectomy, venous cut down, and laparotomy; all producing similar hypocitricemic effects. Thus, the human and animal studies reveal that hypocitricemia is a common condition that always occurs in adults following surgery. No information exists regarding children.

## The clinical relevance of surgical postoperative development of hypocitricemia

Since sustained hypocitricemia can have pathophysiological and clinical effects, the issue is whether or not such consequences appear in the hypocitricemic patients during recovery from surgery. Typical potential adverse effects of hypocitricemia (such as decreased urinary citrate and nephrolithiasis; metabolic acidosis) are not common in patients following surgery. We were not made aware of any adverse complications during the recovery period of any of the patients; but this was not a focus of our studies. Moreover, the studies showed that post-op hypocitricemic animals did not exhibit any overt impaired activities (such as food and water consumption, physical activities) that differed from the control animals. Therefore, it is highly likely that the surgical hypocitricemia does not generally introduce any adverse consequences in patients following surgery.

Consequently, the hypocitricemia must be associated with a beneficial physiological response to surgery (as we describe below). This is worthy of consideration in light of current issues of surgical intervention, which induces serious and complicated cellular and systemic physiological and metabolic disturbances and consequences.^9–12^ As such, the postoperative management of the patient should consider the importance of maintenance and/or restoration of homeostasis of the normal body fluid composition. As noted by Awad and Lobo;^9^ “The past decade has witnessed a paradigm shift in the management of patients undergoing elective surgery, specifically with respect to…implementation of enhanced recovery protocols and perioperative optimization of nutritional status and metabolic function.” Similarly, Evans et al.,^10^ state, “Effective nutrition supplementation and metabolic manipulation have the potential to enhance effective cell metabolism to ensure a successful stress response for the cell and ultimately improve surgical outcomes.”

Obviously, surgical hypocitricemic relationships that we are describing can have important metabolic implications associated with the post-op period; which should be considered in relation to the preceding comments. For example, it is now evident that liver clearance and increased hepatocyte uptake and metabolic utilization of plasma citrate constitute a major response that results in hypocitricemia following surgery. Consequently, consideration of a dietary source for plasma citrate concentration could be important for maintaining the liver response; which is likely involved in the patient recovery from surgery.

## Increased liver clearance and hepatocyte transport and metabolic utilization of plasma citrate; the major factor in the hypocitricemic response to surgery

The renal clearance and excretion of citrate is generally the major process for the removal of citrate from plasma. However, the hypocitricemia in patients did not exhibit any change in urinary excretion of citrate. Rat studies^6^ also revealed that there is no change in the renal clearance of plasma citrate involved with the surgical hypocitricemia (Table 3); which is consistent with the human studies.

**Figure 4:**
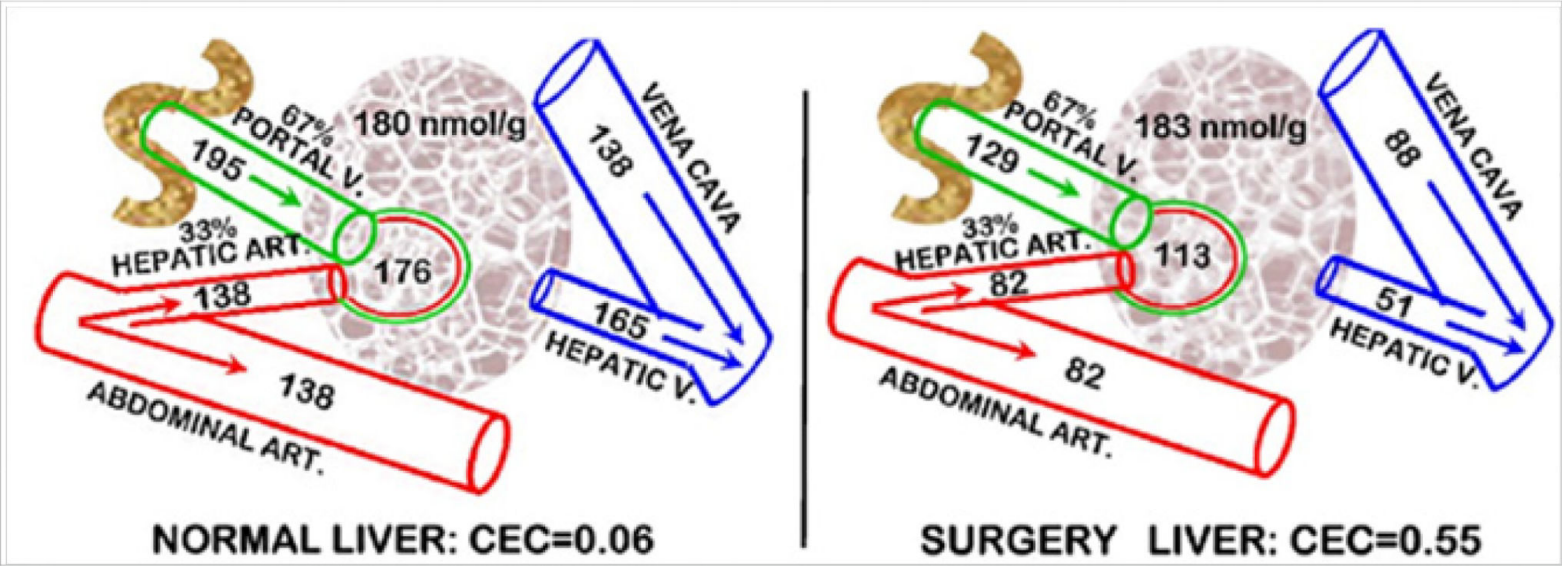
Hepatic citrate concentrations and the clearance of citrate following surgery in the rat. For humans the arterial and portal vein are 25% and 75%, respectively. Plasma and tissue citrate concentrations are uM. **Abbreviation:** CEC, citrate extraction coefficient

Thus, an alternative process is involved in achieving surgical hypocitricemia, which caused us to consider the possible involvement of the liver. A long-held prevailing view has existed that the liver, under normal conditions, exhibits significant clearance of plasma citrate; which involves hepatocyte transport and metabolic utilization of citrate from plasma. However, no physiological in vivo evidence had established that capability and function of the liver and hepatocytes.^8^ Consequently, we addressed this issue in the rat studies.^7^

(Table 4) presents the determination of liver citrate values in control and surgical animals, which were employed to calculate the liver clearance of plasma citrate.^7^ In rats, the blood supply to the liver is ~67% and 33% from the hepatic portal vein and hepatic artery, respectively (~75% and ~25% in humans) as represented in Figure 4. The results demonstrate that the liver under normal conditions exhibits little, if any, extraction of plasma citrate. However, under conditions following surgery, the liver exhibits ~55% extraction of citrate from plasma. This is the first and (as best that we could determine) only study of liver clearance of citrate under *in vivo* conditions.

Compared to the normal renal clearance of citrate (~40%, Table 3), and consideration of the ~40% greater blood flow through the liver; the clearance of citrate by the liver following surgery is ~2-fold greater than renal clearance of citrate. In addition, the 55% liver extraction of plasma citrate provides ~165nmols citrate/minute for hepatocyte uptake. Since the liver concentration of citrate did not change, the hepatocytes rapidly metabolize the transported plasma citrate. This major utilization of citrate requires studies to establish the metabolic pathway and role of the hepatocyte utilization of the plasma citrate in response to surgery.

It also becomes evident that the hepatocytes under normal conditions exhibit minimal, if any, capability to obtain citrate from plasma. This is typical of most mammalian cells since citrate in plasma exists as trivalent and divalent anions, which do not permeate the cellular plasma membrane. Therefore, the hepatocyte response to surgery must include the up regulation of a plasma membrane citrate uptake transporter (most likely *NaCT*; *Slc13A5*).^8^

The absence of evidence of pathophysiological consequences of hypocitricemia following surgery in patients and in animals leads to the expectation that the hypocitricemia is associated with a beneficial physiological/metabolic response to surgery. The relationships described make it most likely that increased liver clearance and hepatocyte utilization of plasma citrate, in some (unknown) way, facilitates the patient recovery following surgery. These are important liver relationships that had never been recognized.

## The hormonal factor(s) associated with the hypocitricemic response to surgery

The hepatic hypocitricemic response occurs via an endocrine hormone response to surgical intervention in tissues. It is not known which factor(s) (neurogenic or chemical) results in a citricemic hormone response that induces the hepatic response and hypocitricemia.

Our human and rat studies provide compelling evidence that neither parathyroid hormone (the major hypercitricemic hormone) nor calcitonin (the major hypocitricemic hormone) is responsible for the surgical hypocitricemia. The studies also revealed that glucocorticoids are not associated with the surgical hypocitricemia. Also, the hypocitricemia following surgery in rats is not accompanied by any change in plasma glucose or plasma pyruvate; and only a slight change in plasma lactate (Table 5).^6^ Thus, hormonal factors such as glucocorticoids, insulin, glucagon, and catecholamine, are not likely implicated in the cause of the surgical hypocitricemia. These observations, couple with the exceptionally pronounced hypocitricemic effect causes us to conclude that an unidentified putative hypocitricemic hormone is a likely cause of surgical hypocitricemia.

## The impact of the surgery hypocitricemia relationships on other clinical and biomedical research issues

Any clinical and experimental studies that involve surgical intervention must consider the presence and impact of surgery hypocitricemic relationships. This will likely require the inclusion of a surgery control group. Without this, the effects of the studies will not identify and differentiate the hypocitricemic effects of surgery from the issue and effects that are the focus of the investigation, and raises questions of the validity of the conclusions and the translational application of the results of the investigation.

In this regard, an important issue is the process of liver regeneration following major liver damage, liver tissue resection and transplantation. The essential early event is the repopulation of the hepatocytes in the regenerating liver. This necessitates the transition of the residual normal functioning hepatocytes to highly proliferating hepatocytes, followed by the transition of the proliferating hepatocytes to normal functioning hepatocytes. The hepatocytes undergo “genetic/ metabolic transformations” from their normal cellular metabolism to extensive lipid biosynthesis, which is the earliest metabolic event in the initiation of the regeneration process.^13–15^ In order to establish the genetic/metabolic changes specifically associated with the liver regeneration process, there must be a control for the hypocitricemic effects of surgery; especially since citrate metabolism is of major importance for lipid biosynthesis. However, no such studies have included a surgical hypocitricemic control, which now requires new studies to validate or challenge the contemporary view and understanding of the metabolic implications in liver regeneration.

In fact, the central role of citrate metabolism in extensive de novo lipid biosynthesis applies to all highly proliferating cells; such as in embryogenesis, stem cells for differentiation, malignant cells during tumorigenesis; and when necessary, such studies must consider the possible impact of surgical hypocitricemia.

## Summary of the clinical relevance of surgical post-op development of hypocitricemia

The physiological and metabolic status of surgery patients and their postoperative recovery has received much attention in recent years. Although sustained hypocitricemia often represents or imposes pathophysiological consequences, the post–operative hypocitricemia and the major involvement of liver clearance and hepatocyte utilization of plasma citrate likely represents a physiologic/metabolic response, which result in hypocitricemia.

That hepatic increased clearance of citrate and not renal clearance of citrate is a major factor in the removal of citrate from circulation is a new understanding of citrate homeostasis and a new role of liver function. Hepatocyte citrate-related metabolism has implications in many pathways of intermediary metabolism, including interactions with other tissues and organs. Consequently, it is highly plausible to suggest that extensive hepatocyte utilization of plasma citrate represents a metabolic transformation in support of the recovery from surgery. If so, dietary citrate supplement might be of benefit for the patient recovery process. Such clinical implications should be important considerations.

Despite its clinical relevance, the hypocitricemic response to surgery, along with the involvement and role of the liver and altered hepatocyte metabolism has been unrecognized and/or ignored by the medical community for about forty years. Hopefully, this comprehensive review will bring attention to the existence and important implications of surgical hypocitricemia; which will generate interest and support for the needed clinical and experimental research to address the many issues that still exist. This is in the best interest of the medical community and the public-at-large.

## Figures and Tables

**Table 1:** Plasma citrate, cortisol, and calcium in patients following surgery

	Preoperative		Postoperative		
	
	1day	6hours	1day	2–4days	5–7days
Citrate-uM (30)	112 (6)	116 (8)	91 (7)*	76 (4)*	78 (4)*
Cortisol-ug% (30)	18 (2)	57 (5)*	32 (3)	24 (2)	21 (2)
Calcium-mg% (14)	10.2 (0.2)	9.3 (0.2)*	9.4 (0.3)	9.5 (0.3)	9.7 (0.3)

(N)=number of patients; Values are mean (sem);

* P<0.05 vs 1day Preoperative.

**Table 2:** Hypocitricemia following surgery (laparotomy) in rats

Post-op hours	Strain	Control	Surgery
6	W (4)	149 ± 7	139 ± 8
16–20	W (8)	156 ± 6	88 ± 8*
48	W (2)	140 ± 7	119 ± 7*
72	W (2)	150 ± 8	130 ± 6
20	SD(2)	130 ± 8	70± 8*

W=Wistar rats; SD=Sprague-Dawley rats; (N) = experiments.

Each group includes 12 rats. Values are mean ± sem;

* P<0.05 Surgery vs Control

**Table 3:** Renal clearance of citrate following surgery in rats

Post- oh		Plasma citrate (uM)			Urine citrate (umols)
	
hours		Renal artery	Renal vein	A-V/A	
6	Surgery (12)	156 ± 5	94±8	0.40±0.03	
	Control (12)	152 ± 8	94±6	0.38±0.03	
16	Surgery (12)	67 ± 8*	45±6*	0.33±0.02	221±28
	Control (12)	155 ± 6	95±3	0.38±0.03	211±34

(12)= number animals/group; A-V/A=clearance coefficient;

* P<0.05 Surgery vs Control.

**Table 4:** Hepatic citrate changes at 12hours following surgery in rats

Citrate concentration (uM)							

Hepatic artery		Hepatic vein		Vena cava		Liver tissue	

Control	Surgery	Control	Surgery	Control	Surgery	Control	Surgery
138 ± 11	82 ± 5*	165 ± 11	51 ± 7*	195 ± 10	129 ± 10*	180	183
−40 ± 6%		−68 ± 5%		−34 ± 7%		Nil	

% is the change between the Control and Surgery groups.

Values are the mean ± sem from 3 experiments with 12 rats/group:

* P<0.05 Surgery vs Control.

**Table 5:** Plasma metabolite changes in rats at 12 hours following surgery

Metabolite	Group	Arterial	Hepatic vein	Portal vein
Pyruvate	Control	186 ± 17	183 ± 11	227 ± 18
	Surgery	156 ± 12	178 ± 13	242 ± 13
Lactate	Control	1,505 ± 170	3,071 ± 219	2,894 ± 179
	Surgery	1,945 ± 137*	3,330 ± 272	3,545 ± 178*
Glucose	Control	10,801 ± 288	12,763 ± 650	10,584 ± 345
	Surgery	10,930 ± 232	12,386 ± 654	10,729 ± 358
Citrate	Control	135 ± 5	158 ± 9	179 ± 6
	Surgery	98 ± 11*	57 ± 7*	114 ± 12*
